# Interaction of Crohn's Disease Susceptibility Genes in an Australian Paediatric Cohort

**DOI:** 10.1371/journal.pone.0015376

**Published:** 2010-11-08

**Authors:** Josef Wagner, Winnie H. Sim, Justine A. Ellis, Eng K. Ong, Anthony G. Catto-Smith, Donald J. S. Cameron, Ruth F. Bishop, Carl D. Kirkwood

**Affiliations:** 1 Enteric Virus Group, Murdoch Childrens Research Institute, Melbourne, Victoria, Australia; 2 Environmental and Genetic Epidemiology Research, Murdoch Childrens Research Institute, Melbourne, Victoria, Australia; 3 Sequenom Platform Facility, Murdoch Childrens Research Institute, Melbourne, Victoria, Australia; 4 Department of Paediatrics, University of Melbourne, Victoria, Australia; 5 Department of Gastroenterology & Clinical Nutrition, Royal Children's Hospital, Melbourne, Victoria, Australia; Charité-University Medicine Berlin, Germany

## Abstract

Genetic susceptibility is an important contributor to the pathogenesis of Crohn's disease (CD). We investigated multiple CD susceptibility genes in an Australian paediatric onset CD cohort. Newly diagnosed paediatric onset CD patients (n = 72) and controls (n = 98) were genotyped for 34 single nucleotide polymorphisms (SNPs) in 18 genetic loci. Gene-gene interaction analysis, gene-disease phenotype analysis and genetic risk profiling were performed for all SNPs and all genes. Of the 34 SNPs analysed, four polymorphisms on three genes (*NOD2*, *IL23R*, and region 3p21) were significantly associated with CD status (p<0.05). All three CD specific paediatric polymorphisms on *PSMG1* and *TNFRSF6B* showed a trend of association with p<0.1. An additive gene-gene interaction involving *TLR4*, *PSMG1*, *TNFRSF6B* and *IRGM* was identified with CD. Genes involved in microbial processing (*TLR4*, *PSMG1*, *NOD2*) were significantly associated either at the individual level or in gene-gene interactive roles. Colonic disease was significantly associated with disease SNP rs7517847 (*IL23R)* (p<0.05) and colonic and ileal/colonic disease was significantly associated with disease SNP rs125221868 (*IBD5)* and *SLC22A4* & *SLC22A4/5* variants (p<0.05). We were able to demonstrate genetic association of several genes to CD in a paediatric onset cohort. Several of the observed associations have not been reported previously in association with paediatric CD patients. Our findings demonstrate that CD genetic susceptibility in paediatric patients presents as a complex interaction between numerous genes.

## Introduction

Crohn's disease (CD) is a chronic relapsing inflammatory disease occurring anywhere in the gastrointestinal tract, although it most commonly affects the small intestine [1]. CD is a major cause of morbidity throughout the world with an escalating epidemic of CD recorded globally in children and adults during the past few decades [2]. A worldwide study reported an incidence per 100,000 population as low as 0.3 in China to as high as 20.2 cases in Canada [2]. A ten-fold increase in the incidence of paediatric CD over a 31 year period was reported from the Royal Children's Hospital (RCH) in Melbourne, Australia [3]. Approximately 30 new cases of CD in children (age 2–16 years) are now diagnosed and treated at the RCH each year compared with approximately 3 new cases reported annually in 1975. European studies report a similar dramatic increase in the incidence of paediatric CD [4], [5]. It is widely accepted that CD is mediated by a dysfunctional immunological response of T-lymphocytes which is primarily induced in genetically susceptible individuals by the presence of an environmental stimulus [6], [7].

Genetic factors that affect susceptibility to CD have been identified using genetic linkage and population based association studies. Genetic susceptibility to CD has been extensively studied since the identification of the first CD susceptibility gene *NOD2* [8], [9]. The NOD gene family is proposed to function as an intracellular pattern-recognition receptor that senses microbial muramyl dipeptide, a degradation product of peptidoglycan from bacterial cell wall and the function of a cytosolic sensor for the induction of apoptosis [10]. In the last decade several genome-wide association studies (GWAS) have discovered an increasing number of novel genes and single nucleotide polymorphisms (SNPs) associated with CD, including 21 novel loci identified in 2008 alone [11].

Paediatric-onset CD patients have a higher rate of gene mutations compared with adult patients [12]. Three mutations were reported to be specifically associated with paediatric-onset inflammatory bowel disease. One of these (rs2836878) resides in a region that harbours no gene, but is most closely located to the proteasome assembly chaperone 1 gene (*PSMG1*). The other two SNPs (rs4809330 and rs2315008) are located within a region containing several genes including the tumour necrosis factor receptor superfamily member 6B gene (*TNFRSF6B*) [13]. All three paediatric specific CD mutations were recently confirmed to be associated with CD in a Canadian study [14].

The large number of genetic variants implicated in CD requires multiple SNPs to be investigated simultaneously in CD patients to understand the individual contributions of loci in single genes and gene-gene interactions. In our study we investigated the occurrence of 34 SNPs simultaneously in a paediatric onset CD cohort.

## Results

### Hardy-Weinberg equilibrium testing

Three SNPs (rs2836878, rs2066845 and rs5743289 present on *PSMG1* and *NOD2*) were found to deviate from Hardy Weinberg Equilibrium (HWE) at the P = 0.05 level. However, these SNPs were retained in the analyses as in each case the deviation from HWE was observed in the cases only. Such distortions in case genotype frequency can be an indication of association [15], [16]. Thus, alleles and genotypes from all SNPs were compared between the case and control phenotypic groups.

### Genotype and allele frequencies

All SNPs were initially analysed using allelic and genotypic χ^2^ tests (Table S 1). For SNPs where the minor allele homozygote counts were less than 5 (rs11209026, rs3792876, rs13361189, rs6958571, rs17327442, rs4986790, rs2241136, rs2289310, rs1248696, rs1793004, rs3135932, rs2066844, rs2066845, rs5743289, rs5743293, rs2836878), we applied Fisher's exact test to obtain a genotypic P value. Four SNPs, rs2066845 and rs5743289 on *NOD2* gene, rs11209026 on *IL23R* gene, and rs9858542 on region 3p21, demonstrated evidence of association with CD (Table 1 and Table S1).

**Table 1:** Genotypic distribution of CD associated genetic variants.

Gene-SNP	Genotype	CD		Controls		P = X^2^ or F*	OR (95% CI)
*NOD2*		n	%	n	%		
rs2066845	GG	64	89	98	100		
	**CG+CC**	**8**	**11**	**0**	**0**	0.00081*	
rs5743289	CC	42	59	65	66		
	CT	20	28	33	34		
	**TT**	**9**	**13**	**0**	**0**	0.00039*	

Main *NOD2*		n	%	n	%		
	GG/CC/DEL	51	72	87	89		
		**20**	**28**	**11**	**11**	**0.005**	3.12 (1.39–6.90)

*IL23R*		n	%	n	%		
rs11209026	GG	68	94	83	85		
	**AG**	**3**	**6**	**15**	**15**	0.023*	0.325 (0.11–0.98)
	AA	0	0	0	0		

3p21		n	%	n	%		
rs9858542	GG	24	34	56	57		
	**GA+AA**	**46**	**66**	**42**	**43**	0.003*	2.56 (1.36–4.81)

*PSMG1*		n	%	n	%		
rs2836878	GG	32	46	55	59		
	**AG+AA**	**38**	**54**	**38**	**41**	**0.089**	1.72 (0.92–3.21)

*TNFRSF6B*		n	%	n	%		
rs4809330	GG	40	56	42	43		
	**AG+AA**	**31**	**44**	**56**	**57**	**0.083**	1.72 (0.93–3.18
rs2315008	GG	40	56	42	43		
	**GT+TT**	**30**	**44**	**56**	**57**	**0.068**	1.78 (0.96–3.30)

Four SNPs on three genes (*NOD2*, *IL23R* and 3p21 region) were significantly associated with paediatric onset Crohn's disease (CD) (p<0.05). Main *NOD2* variants = SNP rs2066845, SNP rs2066844, rs5743293. Three SNPs on two paediatric specific CD susceptibility genes (*PSMG1* and *TNFRSF6B*) showed a trend of association (p<0.1), X^2^ = Pearson Chi Square analysis, F = Fisher's exact test analysis, OR = odds ratio, CI = confidence interval.

#### a) Additive and genotypic logistic regression analyses

At the individual level, allelic χ^2^ and genotypic Fisher's comparisons of SNP rs2066845 (*NOD2*) were highly significant at p<0.05 (p = 0.00016, 0.0076 respectively). However, no minor allele homozygotes or heterozygotes were observed for controls, and therefore further analyses by logistic regression were not performed for this SNP.

Allelic χ^2^ and genotypic Fisher's comparisons of SNP rs5743289 (*NOD2*) were significant at p<0.05 (p = 0.027, 0.0088 respectively). This SNP remained associated by additive, but not genotypic, logistic regression (Additive: OR = 1.9; 95% CI 1.1, 3.3; p = 0.020, Genotypic (2df): p = 0.998; OR for heterozygotes compared with major allele homozygotes = 1.02; 95% CI 0.49, 2.1 (p = 0.952), no minor allele homozygotes observed in controls).

Allelic χ^2^ and genotypic Fisher's comparisons of SNP rs11209026 (*IL23R*) were significant at the p<0.05 level (p = 0.025, 0.023 respectively). This SNP remained associated by additive logistic regression (OR = 0.26, 95% CI 0.07–0.97, p = 0.045). Genotypic logistic regression was not performed due to the lack of minor allele homozygotes.

Allelic and genotypic χ^2^ comparisons of SNP rs9858542 on region 3p21 were significant at the p<0.05 level (p = 0.021, 0.010 respectively). This SNP remained associated by additive and genotypic logistic regression (Additive: OR = 1.8; 95% CI 1.1–2.9; p = 0.02, Genotypic (2df): p = 0.0087).

#### b) Further analyses

Further analyses were performed by comparing the disease associated allele distribution between CD patients and controls (Table 1). Eleven percent of CD patients had at least one disease-associated allele of SNP rs2066845 (*NOD2*) compared to none in the control group (p = 0.00081). Thirteen percent of CD patients had both disease associated alleles of SNP rs5743289 (*NOD2*) compared to none in the control group (p = 0.00039). Analysis of all 3 main *NOD2* variants (rs2066845, rs2066844, and rs5743293) revealed that 28% of CD patients had at least one *NOD2* variant compared to 11% in the control group (OR = 3.1, 95% CI 1.39–6.9, p = 0.005). One patient had a triple mutation in the *NOD2* gene (heterozygote for rs2066844 and rs5743293 and homozygote for rs5743289). Three patients had a double mutation in the *NOD2* gene and four patients had a single mutation in the *NOD2* gene. Sixty six percent of CD patients had at least one disease associated allele of SNP 9858542 (3p21) compared to 43% in the control group (OR = 2.56, 95% CI 1.36–4.81, p = 0.003).

Three SNPs on paediatric specific CD susceptibility genes (rs2836878 on *PSMG1* and rs4809330 and rs2315008 on *TNFRSF6B*) demonstrated some evidence of association at the p<0.1 level of significance. Fifty four percent of CD patients had at least one disease associated allele of SNP rs2836878 (*PSMG1*) compared to 41% in the control group (p = 0.089). Disease associated alleles of SNPs rs4809330 and rs2315008 (*TNFRSF6B*) were observed at lower frequency in CD (44%) patients compared to 57% in the control group (p = 0.083 and p = 0.068 for rs4809330 and rs2315008, respectively).

### Gene-gene interaction

**Figure 1:**
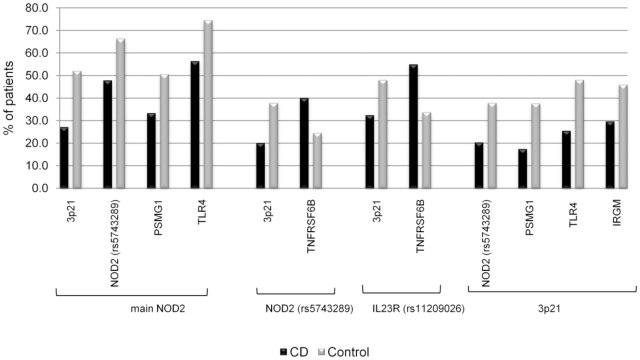
Gene-gene interaction analysis of significant CD associated genes. Percent values represent the proportion of wildtype gene-gene combination. Four wildtype genes were detected in combinations with main *NOD2* wildtype and 3p21 wildtype significantly more often in controls compare to CD patients. *TNFRSF6B* wildtype gene was detected in combination with wildtype SNP rs5743289 (*NOD2*) and wildtype SNP rs11209026 (*IL23R*) significantly more often in CD patients compared to controls. All differences between CD and controls were significant at P<0.05.

Several significant gene-gene interactions were detected for all three disease associated genes (Tables S2a–S2d). There was an under-representation of wildtype allele combinations in CD patients compared to controls harbouring gene combinations of the main *NOD2* variants with four other genetic variants (*PSMG1*, *NOD2* rs5753289, *TLR4*, and 3p21 (Figure 1). There was an under-representation of wildtype allele combinations in CD patients compared to controls harbouring gene combinations of 3p21 variants with four other genetic variants (*PSMG1*, *NOD2* rs5753289, *TLR4*, and *IRGM*) (Figure 1). Interestingly, *PSMG1*, *TLR4*, and *IRGM* were not associated individually with paediatric CD. Conversely, wildtype allele combinations of *TNFRSF6B variants* with *NOD2* rs5743289 or *IL23R* rs11209026 variants were significantly higher in CD patients compared to controls (Figure 1).

### Genotype-Phenotype interaction

The stratification of CD patients according to phenotype is outlined in Table 2. The majority of patients (76%) had ileal/colonic disease with or without upper gastrointestinal tract involvement. We also looked for possible correlation between genotype and disease location and disease behaviour (Table S3). Four disease SNPs from three genes had significant association with disease location. Disease SNP rs7517847 (*IL23R*) was found more often in CD patients with colonic disease (L2±L4) (14% (10/69)) compared to the wildtype form of this SNP in the same phenotype (1% (1/69)) (p = 0.04) (Table S3). Disease SNP rs12521868 (*IBD5*) was found more often in CD patients with colonic and ileal/colonic disease (L2±L4 & L3±L4) (69% (47/69)) compared to the wildtype form of this SNP in the same phenotype (24% (17/69)) (p = 0.027) (Table S3). Disease SNPs rs3792876 and rs1050152 (*SLC22A4* & *SLC22A4/5*) were found more often in CD patients with L2±L4 & L3±L4 phenotype (71% (48/68)) compared to the wild type form of the SNPs in the same phenotype (22% (15/69)) (p = 0.019) (Table S3).

**Table 2:** Patient phenotype characteristic.

Disease location	Patient number	Disease behaviour	Patient number
L1±4	4 (5.7%)	B1±P	61 (87.1%)
L2±L4	12 (17.1%)	B2±P	6 (8.6%)
L3±L4	53 (75.7%)	B3±P	3 (4.3%)
L4	1 (1.4%)		

L1±L4 = ileal disease with or without upper gastrointestinal tract (GI) involvement.

L2±L4 = colonic disease with or without GI involvement.

L3±L4 = ileal/colonic disease with or without GI involvement.

L4 = upper GI disease.

B1±P = inflammatory appearance with or without perininal (P) disease.

B2±P = stricturing disease with or without P disease.

B3±P = penetrating disease with or without P disease.

### Genetic risk analysis for CD and control patients

We performed a genetic risk analysis using either all CD SNPs, or only SNPs associated with disease in this study. Quantitative analysis of all CD SNPs revealed that the proportions of patients having 7, 8, 11, 15, 16, 18, 19 and 21 CD associated SNPs were higher in the CD group than the control group (Figure S1a), whereas analysis of wildtype alleles revealed that control patients had 19, 20, 21, 22, 24, and 25 alleles (Figure S1b). However, the overall difference between CD and controls was not significant.

**Figure 2:**
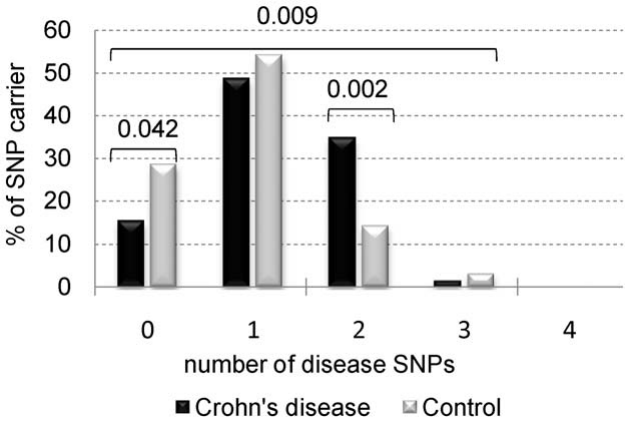
Genetic risk profile analysis of disease associated SNPs between CD patients and controls. The proportion of patients carrying between none and four diseases associated SNPs was calculated for the CD and control group. P values were calculated by Chi Square analysis.

When taking only disease associated SNPs into consideration the overall differences in SNPs between CD patients and controls was significant (p = 0.009) (Figure 2). A significantly lower proportion of CD patients had no disease associated SNPs compared to controls (15.3% versus 28.6%, p = 0.042) whereas, a significantly higher proportion of CD patients had two disease associated SNPs compared to controls (34.7% versus 14%, p = 0.002) (Figure 2 and Table 3).

**Table 3:** Number of disease SNPs associated in CD patients and controls.

Number of SNPs	CD	controls
0	11 (15.3%)	28 (28.6%)
1	35 (48.6%)	53 (54.1%)
2	25 (34.7%)	14 (14.3%)
3	1 (1.4%)	3 (3.1%)

**Figure 3:**
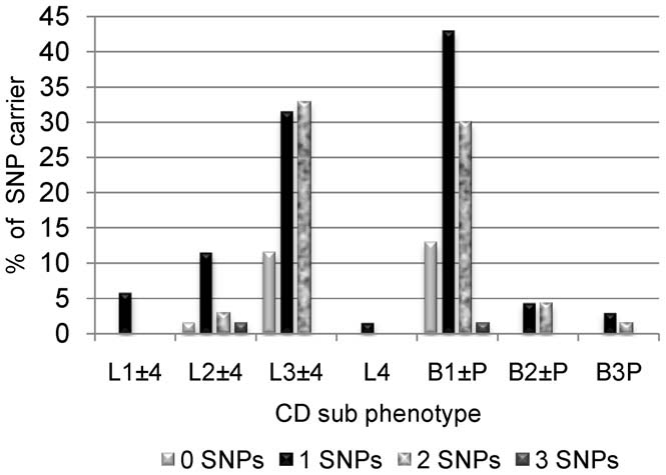
Genetic risk profile analysis stratified by CD phenotypes. The proportion of patients carrying between none and four diseases associated SNPs was stratified by CD phenotype. L1±4 = ileal disease with or without upper gastrointestinal tract (GI) involvement, L2±L4 = colonic disease with or without upper GI involvement, L3±L4 = ileal/colonic disease with or without upper GI involvement, L4 = upper GI disease, B1±P = inflammatory appearance with or without perininal disease, B2±P = stricturing appearance with or without perininal disease, B3P = penetrating appearance with perianal disease.

Genetic risk analysis for CD phenotype showed that one and two SNPs, respectively, were most commonly implicated in disease location “L3±L4” and disease behaviour “B1±P” (Figure 3). Location phenotype analysis revealed that rs9858542 (3p21) SNP was the most common single SNP in the most common location (L3±L4) (63.6%) followed by rs5743289 (*NOD2*) SNP (27.3%) (Table 4). Together these SNPs also represented the most common grouping within the most common phenotype L3+L4 (65.2%) (Table 4). Behaviour phenotype analysis revealed that the rs9858542 (3p21) SNP was the most common single SNP in the most common behaviour group (B1±P) (50%), this was followed by SNP rs5743289 (*NOD2*) (40%) (Table 4). Together these SNPs also represented the most common grouping within the most common behaviour group (B1±P) (71.4%) (Table 4).

**Table 4:** Number of disease SNPs stratified by Crohn's disease phenotype.

	Disease location				Disease behaviour		
Number of SNPs	L1±4	L2±L4	L3±L4	L4	B1±P	B2±P	B3±P
0		1 (1%)	8 (11%)		9 (13%) (43%)		
1	4 (6%)	8 (11%)	22 (31%)^A^	1 (1%)	30 (43%)^C^	3 (4%)	2 (3%)
2		2 (3%)	23 (33%)^B^		21 (30%)^D^	3 (4%)	1 (1%)
3		1 (1%)			1 (1%)		

A = 63.6% 3p21, 27.3% *NOD2* rs5743289, 9.1% *NOD2* rs2066845.

B = 65.2% 3p21 and *NOD2* rs5743289, 30.4% 3p21 and *NOD2* rs2066845, 8.7% 3p21 and *IL23R* rs11209026.

C = 50% 3p21, 40% *NOD2* rs5743289, 6.6% *NOD2* rs2066845, 3.4% *IL23R* rs11209026.

D = 71.4% 3p21 and *NOD2* rs5743289, 23.8% 3p21 and *NOD2* rs2066845, 4.8% 3p21 and *IL23R* rs11209026.

## Discussion

This study analysed a paediatric-onset CD population for the prevalence of 34 SNPs present on 18 genes, to investigate their gene-gene interaction and to perform genetic risk profiling. Four SNP variants present on *NOD2*, *IL23R* and on a 3p21 chromosomal region were significantly associated with our CD population. At the individual level, these SNPs have been reported previously, but no studies have investigated their interaction in a paediatric CD cohort. Three CD specific paediatric SNP variants present on gene *PSMG1* and *TNFRSF6B* were also included in our investigation [13] and while none showed a significant association, all three showed a trend towards association (p<0.1). This represents the second independent confirmation in a case-control study of a possible role for these SNPs in development of CD. There was a higher representation of *PSMG1* SNP variant in CD patients, while a higher representation of *TNFRSF6B* SNP variants was observed for controls. The higher representation of *TNFRSF6B* SNP variants in controls is in contrast to the original study and to a Canadian case/control study [13], [14]. This study also demonstrated that a small patient cohort was sufficient for inferences of CD predisposing gene-gene interactions in association with paediatric-onset disease.

Our combined heterozygous/homozygous detection rate of the main *NOD2* variants in CD patients was 28% compared with 11% in the control group. Previous studies have reported a detection rate between 11%–41% in CD patients and 3–11% in controls [12], [17], [18]. These genetic differences possibly reflect regional and ethnic differences in study populations, highlighted by the virtual absence of *NOD2* variants in a Japanese study [19]. SNP rs5743289 of *NOD2* was previously identified in GWA studies using paediatric and adult cohorts [13], [20]. The significant association of the minor homozygote variant with our CD group confirms the earlier GWAS findings and strengthens the role of *NOD2* in paediatric CD patients. One patient with a triple mutation in the *NOD2* was a 13 year old girl with a L3+4/B1 phenotype. At the time of initial CD diagnosis she had presented with oesophagitis, focal active gastritis, granulomatous colitis consistent with CD in the colon, and chronic active proctitis, however, the role of *NOD2* triple mutation in this multiple disease presentation is not clear.

*IL23R* variants were first described in 2006 [21]. In our study the protective minor A allele of SNP rs11209026 was detected in 15% of controls and in 6% of CD patients. In two previous paediatric studies, the detection rate was 5.5% and 6% in controls and 3% and 2% in CD patients [22], [23]. Intronic *IL23R* SNP variants (rs7517847 and rs1004819) were not associated with our CD group, which is contrary to the Canadian paediatric study [22]. However, colonic disease appeared to be significantly more common in our CD patients with disease associated SNP rs751787 compared to wild type genotype, suggesting that genetic alterations might play a role in CD phenotypic appearance.

The intronic synonymous SNP rs9858542 on 3p21 in close proximity to the *Basson* (BSN) gene was first reported to be associated with CD by the Welcome Trust Case Control Consortium [24]. The significant association of the heterozygote/homozygote genotype identified in our study (66% in CD and 43% in controls) is similar to two other studies (60% and 61.5% in CD and 45% and 52.4% in controls) [25], [26]. We also confirmed the involvement of the minor risk allele (A) as reported by a German and Spanish study [26] but not detected in a US paediatric study [27]. The role of synonymous SNPs in CD should not be underestimated. It was reported that synonymous SNPs can alter mRNA stability, gene expression or can act in linkage disequilibrium with other important SNPs [28].

Gene-gene interaction analysis performed by stratification of disease associated SNPs both within, and between, the candidate genes examined in this study have revealed some very interesting findings. In particular the apparent interaction of *PSMG1* and *TLR4* with the main *NOD2* variants, and *PSMG1*, *TLR4*, and *IRGM* with 3p21, are of interest because these genes were not associated individually with paediatric CD in our cohort (Figure 1). This finding illustrates the complex genetic architecture of CD, in that it is unlikely to be dependent on a single gene but probably is polygenic in nature. A number of genes in combination are likely to affect immunological response and microbial detection, and hence CD risk.

Several of the identified gene-gene variants have been implicated in microbial detection and interaction. Best known is *NOD2*, the product of which is important for the innate recognition of bacterial lipopolysaccharides and peptidoglycans [10], [29], [30]. *TLR4* SNP variant was not associated individually in our CD cohort. However, its significant gene-gene association with *NOD2* and the lipopolysaccharide-signalling role of cell surface toll-like receptors [31], [32], provides strong evidence of a microbial role in CD genetically susceptible individuals.

The role of *PSMG1* SNP variant which showed a trend towards an association with CD, and its significant association with *NOD2* and 3p21 variants suggests an influence on chaperone-driven proteasome assembly which is important in degradation of proteins [33]. Up to a three-fold increase in expression of the proteasome subunit (LMP2), which plays a role in the formation of immunoproteasome, has been reported in the inflamed gut of patients with CD and ulcerative colitis [34], [35]. Bacterial lipopolysaccharides have been shown to trigger the formation of immunoproteasomes *in vivo* mouse cell culture models [36] and play a role in the generation of active NF-kappaB subunits [37], [38]. The significantly larger double mutation rate of *NOD2* and *PSMG1* variants in the CD group (60%) compared with the control group (18%) may also suggest that *NOD2* mutations affect NF-kappaB signalling and *PSMG1* mutations may potentiate microbial-triggered inflammation in CD patients.

*IRGM* gene expression regulates cellular autophagy of internalized bacteria, a process implicated in CD [39]. Studies have shown that *IRGM* gene mutation was not confirmed in children but in ileal CD in the adult population [40]–[42]. However, its role in gene-gene association with 3p21 as identified in our study requires further evaluation. A recent study reported an association between 3p21 variants and another variant of IRGM gene (rs10000113) which was not investigated in our study [43].

Another interesting finding of our study is the negative association of *TNFRSF6B* mutations with CD. The trend towards association with controls at the individual level and significant associations with SNPs rs5743289 (*NOD2*) and rs11209026 (*IL23R*) in controls; point towards a possible role in protection against development of CD. The role of mutations in tumour necrosis factor receptor genes in CD is not well known. A case/control study investigating genetic variants of *TNFRSF1A* and *1B* in association with the three main *NOD2* variants, reported that one out of two SNPs from each gene was significantly implicated in the CD cohort [44].

The non-significant differences considering all SNPs, between CD patients and controls, demonstrate that large genotypic variation occurs in the general population. As a result, a clear IBD wildtype genotype is difficult to define, but is rather a mixture of major homozygote and heterozygote combination.

This study illustrates that an association between the number of SNPs and disease status can be established. A high occurrence of SNP rs9858542 (3p21) with ileal/colonic disease and inflammatory behaviour with SNP rs5743289 (*NOD2*) were identified. Other studies have been equivocal about the association of rs9858542 with CD phenotype [25], [26].

The advantage of this study is that our analysis combined 34 CD susceptibility SNPs in a single paediatric onset cohort. As a result we report novel findings of associations between disease-associated SNPs and paediatric CD phenotypes. A limitation of this study is the relatively small sample size compared to adult studies. Currently, we are not able to repeat the study by recruiting another paediatric cohort to confirm our findings.

In conclusion, this study has shown that CD susceptibility genes are likely act in a complex interactive manner in paediatric-onset CD. Several genes involved in microbial processing (*TLR4*, *PSMG1*, *NOD2*) were significantly associated either at the individual level or synergistically with other genes. A possible novel protective effect of *TNFRSF6B* genetic variants, in combination with two other genes, was suggested however, this was not confirmed by a larger cohort study [14]. Many of the genetic interactions identified have not been reported previously. The results are important to understanding the pathogenesis of CD, however, need to be confirmed in future studies.

## Methods

### Study population

In this study 72 paediatric CD patients and 98 paediatric control patients were analysed. All patients were admitted through the Department of Gastroenterology at the Royal Children's Hospital, Melbourne, Australia. The clinical diagnosis of CD was established using standard clinical, endoscopic, and histopathological criteria according to the Montreal classification [45]. Patients in the control group had been admitted for investigation of symptoms of inflammatory bowel disease (IBD) but were diagnosed either with gastritis, oesophagitis or no pathological condition. All the patients were recruited at initial diagnosis. UC patients were not included in this study due to the low number of patients available. The mean age in the CD group and control group were 11.6 years (2.2–17.2) and 11.9 years (1.7–19.8), respectively. The male/female ratio in the CD group and control group was 46/26 and 45/53, respectively. The CD phenotype subgroups are present in Table 2.

### Ethics Statement

Ethics approval for the study was obtained from the Human Research Ethics Committee of the Royal Children's Hospital (EHRC no.23003). Written informed consent was obtained from each individual, parent or guardian prior to enrolment in the study.

### Genotype Analysis

34 SNPs from 18 genes were selected for analysis. The SNPs and genes were selected from published data that implicated these SNPs in children and/or adults (Table 5).

**Table 5:** Genes and SNPs analysed in this study.

Gene	SNP	Function (Chr No)	−/−	+/+	+/−	Study
*PSMG1*	rs2836878	Intron (21)	GG	AA	AG	children only [13], [14]
*TNFRSF6B*	rs4809330	Intron (20)	GG	AA	AG	children only [13], [14]
	rs2315008	Intron (20)	GG	TT	GT	children only [13], [14]
*NOD2*	rs2066844	Missense Arg702Trp (16)	CC	TT	CT	children and adult [12], children [40], [51], adult [8], [52]–[55]
	rs2066845	Missense Gly908Arg (16)	GG	CC	CG	children [40], [51], adult [8], [52]–[55]
	rs5743293	Frame shift Leu1007FsinsC (16)	DEL/DEL	CC	C/DEL	children [40], [51], adult [8], [52]–[56]
	rs5743289	Intron (16)	CC	TT	CT	children [13], adult [20]
*NOD1*	rs6958571	Intron (7)	AA	CC	CA	adult [57]
*IL23R*	rs1004819	Intron (1)	CC	TT	CT	children [22], adult [21], [58] ^7 = GW^
	rs7517847	Intron (1)	TT	GG	GT	children [22], children and adult [59], adult [21], [58] ^7 = GW^
	rs11209026	Missense Arg381Gln (1)	GG	AA	AG	children [22] [27], children and adult [59], adult [21], [58] ^7 = GW^
*IL10RA*	rs2229113	Missense Arg351Gly (11)	GG	AA	AG	adult [60]*,
	rs3135932	Missense Ser159Gly (11)	AA	GG	GA	adult [60]*,
*DLG5*	rs2289310	Missense Pro1481Gln (10)	CC	AA	CA	adult [61] ^GW^*, adult [61], [62], adult [63]*
	rs1248696	Missense Gln140Pro or Gln140Arg (10)	CC	TT	TC	children and adult [12]*, adult [62]–[64]*, adult [61], [62]
	rs1270912	Intron (10)	GG	AA	AG	children and adult [12]*, adult [61] ^GW^*
	rs2289311	Intron (10)	CC	TT	CT	adult [63], [64]*, adult [61] ^GW^, adult [62]
	rs2165047	UTR-3 (10)	GG	AA	AG	children and adult [12]**, adult [61] ^GW^
	rs1344966	(10)	AA	GG	GA	adult [61] ^GW^
*IBD5*	rs11739135	(5)	GG	CC	GC	adult [65]–[67], children [68]
	rs12521868	Intron (5)	GG	TT	GT	adult [55], [65]–[67], children [68]
*SLC22A4*	rs3792876	Intron (5)	CC	TT	TC	children and adult [12]***
*SLC22A4*	rs1050152	Missense Leu503Phe (5)	CC	TT	TC	adult [55], [67], [69]
*ATG16L1*	rs2241880	Missense Thr281Ala & Thr300Ala (2)	CC	TT	CT	children [27] [40], children and adult [59]**, adult [70], adult [71], adult [72] ^GW^
*IRGM*	rs13361189	(5)	TT	CC	TC	children [27], adult [73] ^GW^
*NKX2-3*	rs10883365	(10)	GG	AA	GA	children [27], adult [73] ^GW^, adult [74]–[76]
3p21 *Basson*	rs9858542	Intron (3) Synonymous Thr3912Thr	GG	AA	GA	children [27], adult [26], children and adult [25].
10q21.1	rs224136	(10)	CC	TT	CT	children [27]
*TLR4*	rs4986790	Missense Asp299Gly (9)	AA	GG	GA	children and adult [12]**, adult [53], [54]
*NELL1*	rs1793004	Intron (11)	CC	GG	CG	adult [77], children [27]*
*ABCB1*	rs17327442	Intron (7)	TT	AA	AT	children [78]
*MYO9B*	rs2305764	Intron (19)	CC	TT	TC	adult [79]
	rs1545620	Missense Ser1011Ala (19)	AA	CC	CA	adult [79]
	rs962917	Intron (19)	CC	TT	TC	adult [79]

Thirty four SNPs from 18 genes were included in this study. (−/−) = major homozygote genotype, (+/+) = minor homozygote genotype, (+/−) = heterozygote genotype, GW = genome wide association studies, * = no significance reported, ** = only reported to be significant and adult, *** = only reported to be significant in children. Chr No = chromosome number. If not otherwise indicated, all studies described here reported a significant association of the described SNP in either genotype frequency, allelic frequency or both.

Genomic DNA was extracted from gut biopsies or blood according to protocols in our laboratory [46], [47]. The SNP site flanking regions were retrieved from NCBI SNP reference assembly database (Build 131). The Sequenom genotyping tools (www.mysequenom.com) designed PCR amplification, extension primers and grouped the 34 SNPs into two multiplex assays - 19 multiplex and 16 multiplex assays. The multiplex PCR and extension reactions were carried out using the Sequenom iPLEX Gold reaction protocol. Genotyping was performed using the matrix-assisted laser desorption ionization time-of-flight (MALDI TOF) mass spectrometry platform [48]. Briefly, the assays were performed in 5 µl volume and contained 1 µl DNA (10–20ng), 1× PCR buffer, 2mM MgCl_2_, 500 uM dNTPs, 100nM of PCR primer mix, 0.5 unit PCR enzyme and nuclease free water. The PCR cycling conditions were: 94°C for 4 minutes, and 45 cycles at 94°C for 20 seconds, 56°C for 30 seconds, and 72°C for 1 minute, and a final extension of 72°C for 3 minutes. The second extension PCR reaction was performed by adding 2 µl iPLEX Gold reaction mix to the cleaned up primary PCR product. The iPLEX termination mix contained 0.2 µl iPLEX buffer plus 0.2 µl iPLEX termination mix, 0.94 µl extension primer mix, 0.041 µl iPLEX enzyme, and nuclease free water. The iPLEX cycling conditions were: 94°C for 30 seconds, and 40 cycles at 94°C for 5 seconds followed by 5 cycles at 52°C for 5 seconds and 80°C for 5 seconds. The final extension was at 72°C for 3 minutes. The iPLEX Gold reaction was purified up by adding 16 µl of nuclease free water and 6 mg resin to each well of the 384 well plates and rotated for 5 minutes and centrifuged at 3200 g for 5 minutes. The products were then transferred to a Sequenom SpectroCHIP and analysed on a MALDI-TOF mass spectrometer (Sequenom MassARRAY) and the SNP calls were viewed using the MassARRAY TYPER 4.05 analyser software. In the majority of SNPs (32/34) a base call was obtained for at least 99% CD samples and control samples. Eighty sequencing reactions were performed to retrieve missing base calls, mainly from the *TLR4*, *NOD2*, and *DLG5*.

### Statistical Analyses

Statistical analyses were performed using PLINK version 1.05 statistical software package [49] (http://pngu.mgh.harvard.edu/~purcell/plink/). Differences between cases and controls were assessed using allelic and genotypic χ^2^ analyses. The relationship between case/control status and each SNP showing some evidence of association by χ^2^ testing was also considered using additive and genotypic regression methods with adjustment for the covariates age and sex. Gene-gene interaction analysis was calculated using χ^2^ analyses. For this the wildtype form (major homozygote) of any disease associated genes was stratified with wildtype form of all other genes included in this study. Genotype-phenotype interaction analysis was calculated using Fisher exact test. STATA version 11 was used for χ^2^ and Fisher exact test analyses. Where the number was 5 or below 5 Fisher exact test was used. Multiple testing adjustments were not performed because the study is in essence replication of previous studies regarding individual SNPs (not discovery) (Table 5). This study investigated SNPs that had been previously associated with CD, significantly reducing the risk of false positive findings [50].

## Supporting Information

Figure S1 Genetic risk profile analysis in CD patients and controls for all disease associated SNPs. The proportion of patients carrying between the minimum number (n=7) and maximum number of CD associated SNPs (n=21) were calculated for the CD and control group (Figure S1a). Genetic risk profile analysis in CD patients and controls for all wildtype SNPs. The proportion of patients carrying between the minimum number (n=12) and maximum number of wildtype SNPs (n=27) were calculated for the CD and control group (Figure S1b). (PDF)

Table S1 Genotypic and allelic distribution for all SNPs. Genotype (GENO) frequencies expressed as minor allele homozygote/heterozygote/major allele homozygote and allele frequencies expressed as minor allele/major allele for all SNPs are outlined. (PDF)

Table S2 Gene‐gene interaction with main *NOD2* variants, *NOD2* rs5743289 variant, with *IL23R* rs11209026 variant, and 3p21 rs9858542 variant, respectively. (PDF)

Table S3 Genotype frequency in CD cases stratified by CD phenotype. (PDF)
