# Supplementary material for: Interaction of Crohn's Disease Susceptibility Genes in an Australian Paediatric Cohort
Source: PLoS One. 2010 Nov 8;5(11):e15376. doi: 10.1371/journal.pone.0015376 (PMC2975706; doi:10.1371/journal.pone.0015376)

**Figure S1a.** Genetic risk profile analysis for all CD associated SNPs analysed in our study

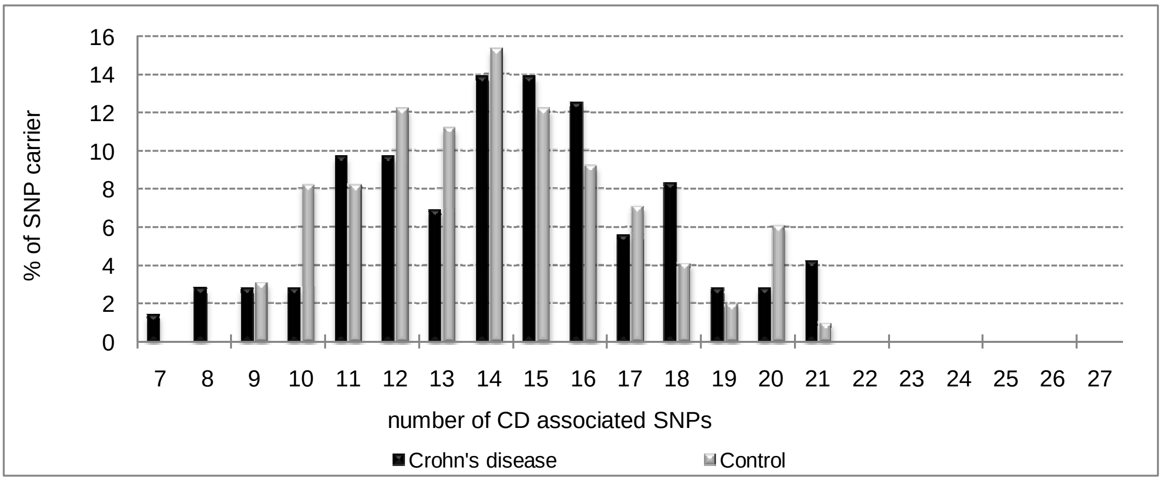

**<u>Figure S1b.</u>** <u>Genetic risk profile analysis for all wildtype SNPs analysed in our study</u>

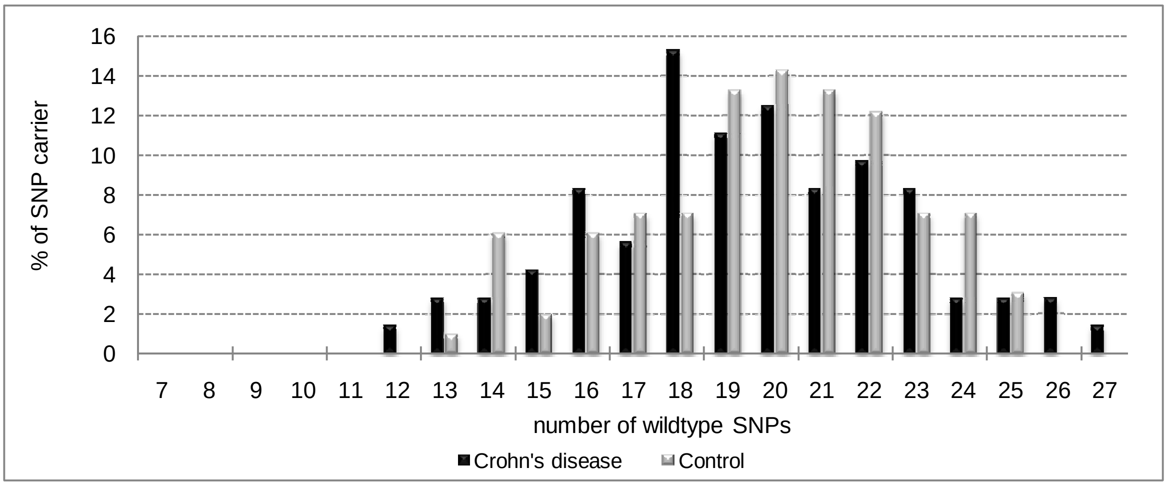

Supplement: Figure S1 — Genetic risk profile analysis in CD patients and controls for all disease associated SNPs. The proportion of patients carrying between the minimum number (n=7) and maximum number of CD associated SNPs (n=21) were calculated for the CD and control group (Figure S1a). Genetic risk profile analysis in CD patients and controls for all wildtype SNPs. The proportion of patients carrying between the minimum number (n=12) and maximum number of wildtype SNPs (n=27) were calculated for the CD and control group (Figure S1b). (PDF) [file pone.0015376.s001.pdf]
