# Supplementary material for: Interaction of Crohn's Disease Susceptibility Genes in an Australian Paediatric Cohort
Source: PLoS One. 2010 Nov 8;5(11):e15376. doi: 10.1371/journal.pone.0015376 (PMC2975706; doi:10.1371/journal.pone.0015376)
Supplement: Table S1 — Genotypic and allelic distribution for all SNPs. Genotype (GENO) frequencies expressed as minor allele homozygote/heterozygote/major allele homozygote and allele frequencies expressed as minor allele/major allele for all SNPs are outlined. (PDF) [file pone.0015376.s002.pdf]

Table S1. Genotypic (GENO) and allelic distribution for all SNPs

CHR	Gene	SNP	minor (risk) allele	major allele	TEST	Crohn's	Control	P X^2 or F*
1	IL23R	rs1004819	T	C	GENO	7/39/25	12/43/43	0.3654
1		rs1004819	T	C	ALLELIC	53/89	67/129	0.5515
1	IL23R	rs7517847	G	T	GENO	6/38/27	16/45/37	0.2942
1		rs7517847	G	T	ALLELIC	50/92	77/119	0.4452
1	IL23R	rs11209026	A	G	GENO	0/3/68	0/15/83	0.02344*
1		rs11209026	A	G	ALLELIC	3/139	15/181	0.02515
2	ATG16L1	rs2241880	T	C	GENO	12/38/21	19/54/25	0.8155
2		rs2241880	T	C	ALLELIC	62/80	92/104	0.5505
3	3p21	rs9858542	A	G	GENO	7/39/24	9/33/56	0.01037
3		rs9858542	A	G	ALLELIC	53/87	51/145	0.02068
5	SLC22A4/5	rs3792876	T	C	GENO	1/7/64	1/11/86	1*
5		rs3792876	T	C	ALLELIC	9/133	13/183	0.9137
5	SLC22A4	rs1050152	T	C	GENO	13/36/22	18/45/35	0.7907
5		rs1050152	T	C	ALLELIC	62/80	81/115	0.668
5	IBD5	rs11739135	C	G	GENO	11/35/25	18/40/39	0.5805
5		rs11739135	C	G	ALLELIC	57/85	76/118	0.8581
5	IBD5	rs12521868	T	G	GENO	12/36/23	18/44/36	0.7538
5		rs12521868	T	G	ALLELIC	60/82	80/116	0.7912
5	IRGM	rs13361189	C	T	GENO	0/13/58	1/16/81	0.9056*
5		rs13361189	C	T	ALLELIC	13/129	18/178	0.9928
7	NOD1	rs6958571	C	A	GENO	4/27/40	6/45/47	0.5863*
7		rs6958571	C	A	ALLELIC	35/107	57/139	0.366
7	ABCB1	rs17327442	A	T	GENO	1/21/49	4/26/68	0.6833*
7		rs17327442	A	T	ALLELIC	23/119	34/162	0.7805
9	TLR4	rs4986790	G	A	GENO	2/11/59	2/12/84	0.759*
9		rs4986790	G	A	ALLELIC	15/127	16/180	0.4505
10	10q21.1	rs2241136	T	C	GENO	2/9/61	2/14/82	0.9365*
10		rs224136	T	C	ALLELIC	13/129	18/178	0.9928
10	DLG5	rs1344966	G	A	GENO	11/27/33	14/41/43	0.8821
10		rs1344966	G	A	ALLELIC	49/93	69/127	0.8944
10	DLG5	rs2165047	A	G	GENO	5/30/36	9/39/50	0.8652
10		rs2165047	A	G	ALLELIC	40/102	57/139	0.8547
10	DLG5	rs2289311	T	C	GENO	11/28/32	17/40/41	0.9028
10		rs2289311	T	C	ALLELIC	50/92	74/122	0.632
10	DLG5	rs2289310	A	C	GENO	0/5/66	0/6/92	1*
10		rs2289310	A	C	ALLELIC	5/137	6/190	0.8141
10	DLG5	rs1270912	A	G	GENO	5/37/28	10/44/44	0.5506
10		rs1270912	A	G	ALLELIC	47/93	64/132	0.8599
10	DLG5	rs1248696	T	C	GENO	0/14/57	0/18/79	0.8454*
10		rs1248696	T	C	ALLELIC	14/128	18/176	0.8578
10	NKX2-3	rs10883365	A	G	GENO	16/32/23	21/51/25	0.5737
10		rs10883365	A	G	ALLELIC	64/78	93/101	0.6028
11	NELL1	rs1793004	G	C	GENO	4/24/43	1/40/57	0.1974*
11		rs1793004	G	C	ALLELIC	32/110	42/154	0.8081

Table S1. continued

11	IL10RA	rs3135932	G	A	GENO	1/16/54	6/23/69	0.3552*
11		rs3135932	G	A	ALLELIC	18/124	35/161	0.196
11	IL10RA	rs2229113	A	G	GENO	6/29/36	14/39/45	0.4984
11		rs2229113	A	G	ALLELIC	41/101	67/129	0.3014
16	NOD2	rs2066844	T	C	GENO	0/12/58	0/9/89	0.1567*
16		rs2066844	T	C	ALLELIC	12/128	9/187	0.1374
16	NOD2	rs2066845	C	G	GENO	2/6/64	0/0/98	0.0007627*
16		rs2066845	C	G	ALLELIC	10/132	0/196	0.0001623
16	NOD2	rs5743289	T	C	GENO	9/20/42	0/33/65	0.0008846*
16		rs5743289	T	C	ALLELIC	38/104	33/163	0.02706
16	NOD2	rs5743293	2	1	GENO	0/1/70	0/2/96	1*
16		rs5743293	2	1	ALLELIC	1/141	2/194	0.7597
19	MYO9B	rs962917	T	C	GENO	14/32/25	18/43/36	0.9632
19		rs962917	T	C	ALLELIC	60/82	79/115	0.7782
19	MYO9B	rs1545620	C	A	GENO	17/33/21	22/43/33	0.853
19		rs1545620	C	A	ALLELIC	67/75	87/109	0.6105
19	MYO9B	rs2305764	T	C	GENO	13/34/24	22/43/33	0.7842
19		rs2305764	T	C	ALLELIC	60/82	87/109	0.696
20	TNFRSF6B	rs2315008	T	G	GENO	5/25/40	10/46/42	0.187
20		rs2315008	T	G	ALLELIC	35/105	66/130	0.08737
20	TNFRSF6B	rs4809330	A	G	GENO	6/25/40	10/46/42	0.2205
20		rs4809330	A	G	ALLELIC	37/105	66/130	0.1332
21	PSMG1	rs2836878	A	G	GENO	2/36/32	5/33/55	0.125*
21		rs2836878	A	G	ALLELIC	40/100	43/143	0.2632

Genotype (GENO) frequencies expressed as minor allele homozygote/heterozygote/major allele homozygote. Allele frequences expressed as minor allele/major allele. Fisher's (F*) exact test P value are used where counts are less than five, X^2 = Chi Square test , 2 (NOD2 rs5743293) = no C allele deleted, 1 = one C allele deleted, Chr = chromosome,
