# Supplementary material for: Interaction of Crohn's Disease Susceptibility Genes in an Australian Paediatric Cohort
Source: PLoS One. 2010 Nov 8;5(11):e15376. doi: 10.1371/journal.pone.0015376 (PMC2975706; doi:10.1371/journal.pone.0015376)
Supplement: Table S2 — Gene‐gene interaction with main NOD2 variants, NOD2 rs5743289 variant, with IL23R rs11209026 variant, and 3p21 rs9858542 variant, respectively. (PDF) [file pone.0015376.s003.pdf]

Table S2a. Gene-gene interaction with main *NOD2* variants

	CD main NOD2 variants				Control main NOD2 variants					
	WT	%	M	%	WT	%	M	%	*P*	OR (95% CI)
PSMG1 WT	**23**	**33.3**	8	11.6	**47**	**50.5**	8	8.6	**0.029**	2.04 (1.1-3.9)
PSMG1 M	26	37.7	12	17.4	36	38.7	2	2.2		
TNFRSF6B combined WT	32	45.7	8	11.4	37	37.8	5	5.1		
TNFRSF6B combined M	19	27.1	11	15.7	50	51.0	6	6.1		
DLG5 combined WT										
DLG5 combined M	51	71.8	71	100.0	87	88.8	98	100.0		
SLC22A combined WT	15	21.4	4	5.7	28	28.6	1	1.0		
SLC22A combined M	35	50.0	16	22.9	59	60.0	10	10.2		
IL23R rs11209026 WT	47	66.2	20	28.2	74	75.5	9	9.2		
IL23R rs11209026 M	4	5.6			13	13.3	2	2.0		
IL23R intronic WT	3	4.2	1	1.4	4	4.1	3	3.0		
IL23R intronic M	48	67.6	19	26.8	83	84.7	8	8.2		
3p21 WT	**19**	**27.1**	5	7.1	**51**	**52.0**	5	5.1	**0.001**	2.9 (1.5-5.6)
3p21 M	32	45.7	14	20.0	36	36.7	6	6.1		
NOD2 rs5743289 WT	**33**	**47.8**	8	11.6	**65**	**66.3**			**0.017**	2.2 (1.1-4.0)
NOD2 rs5743289 M	16	23.2	12	17.4	22	22.4	11	11.2		
NOD1 WT	30	42.3	10	14.1	45	45.9	2	2.0		
NOD1 M	21	29.6	10	14.1	42	42.9	9	9.2		
MYO9B combined WT	12	16.9	9	12.7	28	28.6	2	2.0		
MYO9B combined M	39	54.9	11	15.5	59	60.2	9	9.2		
IL10RA combined WT	27	38.0	7	9.9	42	42.9	2	2.0		
IL10RA combined M	24	33.8	13	18.3	45	45.9	9	9.2		
NELL1 WT	26	36.6	16	22.5	50	51.0	7	7.1		
NELL1 M	25	35.2	4	5.6	37	37.8	4	4.1		
IRGM WT	41	57.7	17	23.9	70	71.4	11	11.2		
IRGM M	10	14.1	3	4.2	17	17.3				
TLR4 WT	**40**	**56.3**	19	26.8	**73**	**74.5**	11	11.2	**0.013**	2.3 (1.2-4.3)
TLR4 M	11	15.5	1	1.4	14	14.3				
ATG16L1 WT	13	18.3	7	9.9	23	23.5	2	2.0		
ATG16L1 M	38	53.5	13	18.3	64	65.3	9	9.2		
10q21.1 WT	44	62.0	16	22.5	73	74.5	9	9.2		
10q21.1 M	7	9.9	4	5.6	14	14.3	2	2.0		
IBD5 combined WT	17	23.9	5	7.0	32	32.7	2	2.0		
IBD5 combined M	34	47.9	15	21.1	55	56.1	9	9.2		
NKX2WT3 WT	17	23.9	5	7.0	22	22.7	2	2.1		
NKX2WT3 M	34	47.9	15	21.1	64	66.0	9	9.3		
ABCB1 WT	35	49.3	15	21.1	60	61.2	8	8.2		
ABCB1 M	16	22.5	5	7.0	27	27.6	3	3.1		

WT = wildtype genotype, M = mutant gentotype

if SNPs are not specified by SNP ID then all SNPs on one genes are present combined

OR = odds ratio, CI = confidence interval

Table S2b. Gene-gene interaction with *NOD2* rs5743289 variant

	CD NOD2 rs5743289				Control NOD2 rs5743289					
	WT	%	M	%	WT	%	M	%	*P*	OR (95% CI)
PSMG1 WT	19	27.1	13	18.5	33	35.5	22	23.5		
PSMG1 M	23	32.8	15	21.4	28	30.1	10	10.7		
TNFRSF6B combined WT	**28**	**40.0**	12	17.1	**24**	**24.5**	18	18.4	**0.032**	0.49 (0.25-0.94)
TNFRSF6B combined M	14	20.0	16	22.9	41	41.8	15	15.3		
DLG5 combined WT										
DLG5 combined M	42	59.2	71	100.0	65	67.0	97	100.0		
SLC22A combined WT	13	18.3	6	8.5	20	20.4	9	9.2		
SLC22A combined M	29	40.8	23	32.4	45	45.9	24	24.5		
IL23R rs11209026 WT	40	56.3	28	39.4	53	54.1	30	30.6		
IL23R rs11209026 M	2	2.8	1	1.4	12	12.2	3	3.1		
IL23R intronic WT	4	5.6			4	4.1	2	2.0		
IL23R intronic M	38	53.5	29	40.8	61	62.2	31	31.6		
3p21 WT	**14**	**20.0**	10	14.3	**37**	**37.8**	19	19.4	**0.014**	2.43 (1.2-4.9)
3p21 M	28	40.0	18	25.7	28	28.6	14	14.3		
NOD1 WT	22	31.0	18	25.4	33	33.7	14	14.3		
NOD1 M	20	28.2	11	15.5	32	32.7	19	19.4		
MYO9B combined WT	12	16.9	8	11.3	19	19.6	11	11.3		
MYO9B combined M	30	42.3	21	29.6	46	47.4	21	21.6		
IL10RA combined WT	20	28.2	15	21.1	29	29.6	15	15.3		
IL10RA combined M	22	31.0	14	19.7	36	36.7	18	18.4		
NELL1 WT	24	33.8	19	26.8	35	35.7	22	22.4		
NELL1 M	18	25.4	10	14.1	30	30.6	11	11.2		
IRGM WT	33	46.5	25	35.2	51	52.0	30	30.6		
IRGM M	9	12.7	4	5.6	14	14.3	3	3.1		
TLR4 WT	34	47.9	24	33.8	54	55.1	30	30.6		
TLR4 M	8	11.3	5	7.0	11	11.2	3	3.1		
ATG16L1 WT	12	16.9	9	12.7	16	16.3	9	9.2		
ATG16L1 M	30	42.3	20	28.2	49	50.0	24	24.5		
10q21.1 WT	38	53.5	22	31.0	55	56.1	27	27.6		
10q21.1 M	4	5.6	7	9.9	10	10.2	6	6.1		
IBD5 combined WT	16	22.5	6	8.5	23	23.7	11	11.3		
IBD5 combined M	26	36.6	23	32.4	41	42.3	22	22.7		
NKX2WT3 WT	13	18.3	10	14.1	13	13.4	12	12.4		
NKX2WT3 M	29	40.8	19	26.8	51	52.6	21	21.6		
ABCB1 WT	26	36.6	23	32.4	45	45.9	23	23.5		
ABCB1 M	16	22.5	2	2.8	20	20.4	10	10.2		

WT = wildtype, M = mutant, WT/M = combined wildtype and/or mutant
if SNPs are not specified by SNP ID then all SNPs on one genes are present combined
OR = odds ratio, CI = confidence interval

Table S2c. Gene-gene interaction with *IL23R* rs11209026 variant

	CD IL23R rs11209026				Control IL23R rs11209026					
	WT	%	M	%	WT	%	M	%	*P*	OR (95% CI)
PSMG1 WT	32	45.7			46	49.5	9	9.7		
PSMG1 M	36	51.4	2	2.8	32	34.4	6	6.5		
TNFRSF6B combined WT	**39**	**54.9**	1	1.4	**33**	**33.7**	9	9.2	**0.006**	2.4 (1.3-4.5)
TNFRSF6B combined M	28	39.4	3	4.2	50	51.5	6	6.1		
DLG5 combined WT										
DLG5 combined M	68	95.8	71	100.0	82	83.7	97	100.0		
SLC22A combined WT	19	26.8		0.0	26	26.5	3	3.1		
SLC22A combined M	49	69.0	3	4.2	57	58.2	12	12.2		
IL23R intronic WT	4	5.6			6	6.1		0.0		
IL23R intronic M	64	90.1	4	5.6	77	78.6	15	15.3		
3p21 WT	**23**	**32.4**	1	1.4	**47**	**48.0**	9	9.2	**0.043**	1.9 (1.0-3.6)
3p21 M	44	62.0	3	4.2	36	36.7	6	6.1		
NOD2 rs5743289 WT	39	55.7	2	2.9	53	54.1	12	12.2		
NOD2 rs5743289 M	28	40.0	1	1.4	30	30.6	3	3.1		
NOD1 WT	39	54.2	2	2.8	43	43.9	4	4.1		
NOD1 M	29	40.3	2	2.8	40	40.8	11	11.2		
MYO9B combined WT	20	27.8	1	1.4	27	27.8	3	3.1		
MYO9B combined M	48	66.7	3	4.2	55	56.7	12	12.4		
IL10RA combined WT	33	45.8	2	2.8	37	37.8	7	7.1		
IL10RA combined M	35	48.6	2	2.8	46	46.9	8	8.2		
NELL1 WT	42	58.3	1	1.4	55	56.1	2	2.0		
NELL1 M	26	36.1	3	4.2	28	28.6	13	13.3		
IRGM WT	55	76.4	4	5.6	67	68.4	14	14.3		
IRGM M	13	18.1		0.0	16	16.3	1	1.0		
TLR4 WT	57	79.2	2	2.8	72	73.5	12	12.2		
TLR4 M	11	15.3	2	2.8	11	11.2	3	3.1		
ATG16L1 WT	20	27.8	1	1.4	19	19.4	6	6.1		
ATG16L1 M	48	66.7	3	4.2	64	65.3	9	9.2		
10q21.1 WT	58	80.6	3	4.2	69	70.4	13	13.3		
10q21.1 M	10	13.9	1	1.4	14	14.3	2	2.0		
IBD5 combined WT	22	30.6			28	28.9	6	6.2		
IBD5 combined M	46	63.9	4	5.6	54	55.7	9	9.3		
NKX2WT3 WT	23	31.9			22	22.7	3	3.1		
NKX2WT3 M	45	62.5	4	5.6	60	61.9	12	12.4		
ABCB1 WT	46	63.9	4	5.6	58	59.2	10	10.2		
ABCB1 M	22	30.6			25	25.5	5	5.1		

WT = wildtype, M = mutant
if SNPs are not specified by SNP ID then all SNPs on one genes are present combined
OR = odds ratio, CI = confidence interval

Table S2d. Gene-gene interaction with 3p21 rs9858542 variant

	CD 3p21				Control 3p21					
	WT	%	M	%	WT	%	M	%	*P*	OR (95% CI)
PSMG1 WT	**12**	**17.4**	19	27.5	**35**	**37.6**	20	21.5	**0.004**	2.9 (1.4-6.1)
PSMG1 M	12	17.4	26	37.7	18	19.4	19	20.4		
TNFRSF6B combined WT	12	16.9	28	39.4	22	22.4	20	20.4		
TNFRSF6B combined M	12	16.9	19	26.8	34	34.7	22	22.4		
DLG5 combined WT										
DLG5 combined M	24	34.3	70	100.0	56	57.1	98	100.0		
SLC22A combined WT	6	8.6	12	17.1	17	17.3	12	12.2		
SLC22A combined M	18	25.7	34	48.6	39	39.8	30	30.6		
IL23R intronic WT	2	2.8	2	2.8	4	4.1	2	2.0		
IL23R intronic M	22	31.0	45	63.4	52	53.1	40	40.8		
NOD2 rs5743289 WT	**14**	**20.3**	27	39.1	**37**	**37.8**	28	28.6	**0.016**	2.4 (1.2-4.8)
NOD2 rs5743289 M	10	14.5	18	26.1	19	19.4	14	14.3		
NOD1 WT	14	19.7	27	38.0	28	28.6	19	19.4		
NOD1 M	10	14.1	20	28.2	28	28.6	23	23.5		
MYO9B combined WT	6	8.5	15	21.1	17	17.5	13	13.4		
MYO9B combined M	18	25.4	32	45.1	38	39.2	29	29.9		
IL10RA combined WT	12	16.9	23	32.4	28	28.6	16	16.3		
IL10RA combined M	12	16.9	24	33.8	28	28.6	26	26.5		
NELL1 WT	15	21.1	27	38.0	33	33.7	24	24.5		
NELL1 M	9	12.7	20	28.2	23	23.5	18	18.4		
IRGM WT	**21**	**29.6**	37	52.1	**45**	**45.9**	36	36.7	**0.032**	2.0 (1.1-3.8)
IRGM M	3	4.2	10	14.1	11	11.2	6	6.1		
TLR4 WT	**18**	**25.4**	40	56.3	**47**	**48.0**	37	37.8	**0.002**	2.8 (1.4-5.4)
TLR4 M	6	8.5	7	9.9	9	9.2	5	5.1		
ATG16L1 WT	8	11.3	13	18.3	17	17.3	8	8.2		
ATG16L1 M	16	22.5	34	47.9	39	39.8	34	34.7		
10q21.1 WT	20	28.2	40	56.3	48	49.0	34	34.7		
10q21.1 M	4	5.6	7	9.9	8	8.2	8	8.2		
IBD5 combined WT	7	9.9	14	19.7	20	20.6	14	14.4		
IBD5 combined M	17	23.9	33	46.5	35	36.1	28	28.9		
NKX2WT3 WT	10	14.1	13	18.3	13	13.4	12	12.4		
NKX2WT3 M	14	19.7	34	47.9	42	43.3	30	30.9		
ABCB1 WT	19	26.8	30	42.3	37	37.8	31	31.6		
ABCB1 M	5	7.0	17	23.9	19	19.4	11	11.2		

WT = wildtype, M = mutant

if SNPs are not specified by SNP ID then all SNPs on one genes are present combined

OR = odds ratio, CI = confidence interval
