# Supplementary material for: Interaction of Crohn's Disease Susceptibility Genes in an Australian Paediatric Cohort
Source: PLoS One. 2010 Nov 8;5(11):e15376. doi: 10.1371/journal.pone.0015376 (PMC2975706; doi:10.1371/journal.pone.0015376)
Supplement: Table S3 — Genotype frequency in CD cases stratified by CD phenotype. (PDF) [file pone.0015376.s004.pdf]

Table S3. Genotype frequency in CD cases stratified by CD phenotype

	**Localisation**									**Behavior**					
	L1 ± L4		L2 ± L4		L3 ± L4		L4			B1 ± P		B2 ± P		B3 ± P	
	n	%	n	%	n	%	n	%	***P (F)***	n	%	n	%	n	%
PSMG1 WT	2	3	5	7	22	33	1	1		25	37	4	6	1	1
PSMG1 M	2	3	5	7	30	45				33	49	2	3	2	3
TNFRSF6B WT	3	4	5	7	29	43				33	49	3	4	1	1
TNFRSF6B M			6	9	24	35	1	1		27	40	2	3	2	3
NOD2 rs2066844 WT	3	4	10	15	42	62	1	1		50	74	4	6	2	3
NOD2 rs2066844 M	1	1	1	1	10	15				9	13	2	3	1	1
NOD2 rs2066845 WT	4	6	11	16	55	80	1	1		53	77	5	7	3	4
NOD2 rs2066845 M					8	12				7	10	1	1		
NOD2 rs5743293 WT	4	6	11	16	52	75	1	1		59	86	6	9	3	4
NOD2 rs5743293 M					1	1				1	1				
NOD2 compound WT	3	4	10	15	34	50	1	1		43	63	3	4	2	3
NOD2 compound M	1	1	1	1	18	26				16	24	3	4	1	1
NOD2 rs5743289 WT	2	3	5	7	31	46	1	1		33	49	4	6	2	3
NOD2 rs5743289 M	2	3	6	9	21	31				26	38	2	3	1	1
NOD1 WT	2	3	8	12	29	42	1	1		36	52	2	3	2	3
NOD1 M	2	3	3	4	24	35				24	35	4	6	1	1
IL23R rs1004819 WT	3	4	4	6	18	26				22	32			3	4
IL23R rs1004819 M	1	1	7	10	35	51	1	1		38	55	1	1	5	7
IL23R rs11209026 WT	4	6	9	13	51	74	1	1		57	83	5	7	3	4
IL23R rs11209026 M			2	3	2	3				3	4	1	1		
IL23R rs7517847 WT			**1**	1	26	38				23	33	1	1	3	4
IL23R rs7517847 M	4	6	**10**	14	27	39	1	1	**0.041**	37	54	5	7		
DLG5 compound WT															
DLG5 compound M	4	6	10	15	53	78	1	1		59	87	6	9	3	4
IBD5 rs11739135 WT	3	4	3	4	17	25				18	26	4	6	1	1
IBD5 rs11739135 M	1	1	8	12	36	53	1	1		42	62	2	3	2	3
IBD5 rs12521868 WT	4	6	**3**	4	**14**	20				17	25	4	6		
IBD5 rs12521868 M			**8**	12	**39**	57	1	1	**0.027**	43	62	2	3	3	4
SLC22A4 & SLC22A4/5 WT	3	4	**3**	4	**12**	18	1	1		14	21	4	6		
SLC22A4 & SLC22A4/5 M	1	1	**8**	12	**40**	59			**0.019**	45	66	2	3	3	4
MYO9B compound WT			2	3	17	25	1	1		17	25	2	3	1	1
MYO9B compound M	4	6	9	13	36	52				43	62	4	6	2	3
IL10RA compound WT	2	3	3	4	28	41				28	41	3	4	2	3
IL10RA compound M	2	3	8	12	25	36	1	1		32	46	3	4	1	1
ATG16L1 WT	1	1	4	6	15	22				18	26			2	3
ATG16L1 M	3	4	7	10	38	55	1	1		42	61	6	9	1	1
10q21.1 WT	4	6	8	12	45	65	1	1		51	74	5	7	2	3
10q21.1 M			3	4	8	12				9	13	1	1	1	1
3p21 WT	1	1	5	7	16	24				22	32				
3p21 M	2	3	6	9	37	54	1	1		38	56	5	7	3	4
NKX2-3 WT	2	3	6	9	13	19				20	29	1	1		
NKX2-3 M	2	3	5	7	40	58	1	1		40	58	5	7	3	4
ABCB1 WT	3	4	8	12	38	55				43	62	4	6	2	3
ABCB1 M	1	1	23	33	15	22	1	1		17	25	2	3	1	1
IRGM WT	3	4	10	14	44	64	1	1		52	75	4	6	2	3
IRGM M	1	1	1	1	9	13				8	12	2	3	1	1

Table S3. continued

	L1 ± L4		L2 ± L4		L3 ± L4		L4			B1 ± P		B2 ± P		B3 ± P	
	n	%	n	%	n	%	n	%	***P (F)***	n	%	n	%	n	%
NELL1 WT	2	3	6	9	31	45	1	1		35	51	3	4	2	3
NELL1 M	2	3	5	7	22	32				25	36	3	4	1	1
TLR4 WT	4	6	6	9	45	65	1	1		47	68	6	9	3	4
TLR4 M			5	7	8	12				13	19				

WT (wildtype) = major homozygote genotype
M (mutant) = heterozygote genotype and minor homogzygote genotype
(F) = p value was calculated using Fisher exact test
L1 ± L4 = ileal disease with or without upper gastrointestinal tract (GI) involvment
L2 ± L4 = colonic disease with or without upper GI involvment
L3 ± L4 = ileal/colonic disease with or without upper GI involvment
L4 = upper GI disease
B1 ± P = inflammotry appearance with or without perininal (P) disease
B2 ± P = stricutring appearance with or without P disease
B3 ± P = penetrating appearance with or without P disease
